# Hemophilia B: A Pain in the Back

**DOI:** 10.7759/cureus.36577

**Published:** 2023-03-23

**Authors:** Apurva Vedire, Gautham Upadrasta, Steven Imburgio, Anmol S Johal, Mohammad A Hossain

**Affiliations:** 1 Internal Medicine, Jersey Shore University Medical Center, Neptune, USA; 2 Neurology, Montefiore Medical Center/Albert Einstein College of Medicine, Bronx, USA; 3 Medicine, Hackensack Meridian School of Medicine, Nutley, USA

**Keywords:** chest wall, back pain, factor ix, hematoma, hemophilia

## Abstract

This case report describes an unusual manifestation of hemophilia B, in the form of a lateral chest wall hematoma. A 27-year-old hemophiliac male was found to have a lateral chest wall hematoma after presenting with back pain associated with localized chest wall swelling. Even more unusual than the location of his hematoma was the absence of any preceding triggers such as a fall or trauma to the area. To our knowledge, this is the first reported case of its kind in a patient with inherited hemophilia B. We believe the reporting of such rare presentations increases awareness of these possibilities and thus aids in the prompt diagnosis and treatment of other similar cases when they are encountered.

## Introduction

Inherited hemophilia B is an X-linked bleeding disorder that is diagnosed in individuals with low factor IX activity levels. Normal levels of activity are considered to be around 50-150%. It can be classified as mild, moderate, and severe based on these levels. Patients with severe hemophilia B have <1% activity of factor IX [1]. This can result in frequent joint or deep muscle bleeding. Without prophylactic treatment, these patients are at a high risk of multiple bleeding episodes every month [1]. We present the case of A 27-year-old male with hemophilia B, who presented with severe back pain with subsequent imaging revealing a spontaneous hematoma in a highly unusual location, his chest wall.

## Case presentation

A 27-year-old male with a past medical history significant for hemophilia B presented to our emergency department (ED) with a complaint of back pain. He stated that the pain, which he quantified as extremely severe, was located in the right scapular region and had started the night before. This pain was associated with localized swelling in that region. He had no other associated weakness, paresthesias, or difficulty taking breaths. He denied any preceding trauma to the area and stated that his symptoms began spontaneously while sleeping. The patient also denied any fever, chills, nausea, vomiting, hematuria, melena, or other symptoms. The patient had not been on any antiplatelet or anticoagulation agents. Since his diagnosis of hemophilia B at birth, he reported multiple intramuscular and intra-articular bleeding episodes requiring multiple fasciotomies, hematoma evacuations, and debridements for soft tissue infections. His prior hematomas had occurred in various locations, including his thigh, arm, knees, and face, and were both spontaneous as well as post-traumatic. He had a history of leaving against medical advice (AMA), was non-compliant with his follow-ups, and did not regularly follow up with a hematologist. He admitted to tobacco and marijuana use, however, he denied any other illicit drug or alcohol use. His family history was significant for similar hemophilia episodes in his brother who was now deceased.

His most recent admission occurred two months prior, for a right thigh hematoma complicated by right lower extremity cellulitis requiring antibiotics for which he left AMA prior to treatment being completed. His last measured factor IX level at this time was <1 (reference range 60-160% normal) and no factor IX inhibitor was detected. On admission to the ED, he was vitally stable with a blood pressure of 119/56 mmHg and a heart rate of 85 BPM and was afebrile on room air. His significant laboratory values are shown in Table 1.

**Table 1:** Laboratory values on admission PTT: Partial thromboplastin time; PT: Prothrombin time; INR: International normalized ratio

Laboratory Finding	Result	Reference Range
Hemoglobin	4.8 g/dL	13.2-17.5 g/dL
PTT	78 seconds	26-39 seconds
PT	14.3 seconds	10.1-13.3 seconds
INR	1.25	0.88-1.15

**Figure 1:**
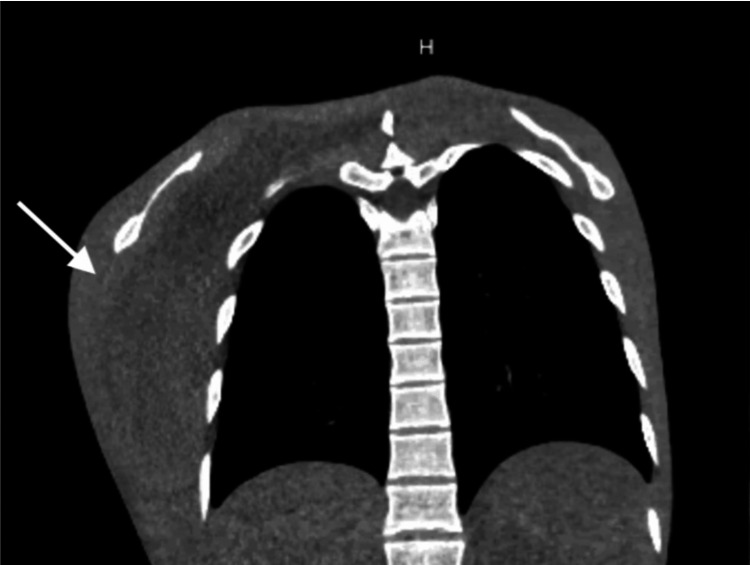
CT scan of the chest (coronal view) Right-sided chest wall hematoma (white arrow)

Given his extensive history of hematomas in the past, a computed tomography scan of the chest and abdomen was completed, which showed a large hematoma in the right lateral chest wall measuring 12.8 x 12.2 x 4.8 cm, along with anasarca (Figure 1).

He was given two units of packed red blood cells, along with two units of fresh frozen plasma (FFP) and recombinant factor IX 100 units/kg. A stat cardiothoracic surgery consult was placed along with a hematology-oncology consult.

The patient completed his transfusions but unfortunately left AMA prior to any evaluation for interventions such as the evacuation of hematoma or angiographic embolization.

## Discussion

Chest wall hematomas are infrequently reported in the medical literature and typically occur in the presence of trauma such as rib fractures. Non-traumatic cases of chest wall hematomas have been increasingly reported with the growing utilization of antiplatelet and anticoagulation agents [2]. Other extremely rare, non-traumatic etiologies, including tumors, arteriovenous malformations, and chronic myeloid leukemia [3], have been reported in scarce case reports. Chest wall hematomas usually have minimal bleeding, as they occur in closed tissue, which allows for compression of the bleeding vessel [4]. However prompt identification is important, as it can sometimes lead to complications such as hemorrhagic shock [4] or compression of adjacent tissue and organs causing hemodynamic instability [5]. 

Joint hemorrhage, mostly in the knees, elbows, and ankles, accounts for 80% of hemorrhages in adult hemophiliacs [6]. Bleeding can also occur in intracranial sites, oropharyngeal sites, and the gastrointestinal and genitourinary tracts. When bleeding involves muscles, it is usually in the large muscle groups such as the quadriceps, iliopsoas, and arms. Extensive bleeding poses a risk for compartment syndrome, especially in the lower leg and forearm. Untreated, it can also lead to the formation of a pseudotumor, where a fibrous membrane surrounds the hematoma [7].

Despite the increased bleeding risk in patients with hemophilia, we identified only a handful of cases of chest wall hematomas in this patient demographic. Two cases reported spontaneous chest wall hematomas, however, both were due to acquired hemophilia A and B, respectively, and not inherited [8,9]. Another reported a case of arm bleeding, which then expanded to the posterior chest wall in a myelodysplastic syndrome patient with acquired hemophilia A [10]. The only case we found of a chest wall hematoma in a congenital hemophilia B patient occurred in the traumatic setting of a fall, with this patient also having coexisting acquired hemophilia A as well [11]. To our knowledge, this is the first reported case of a spontaneous lateral chest wall hematoma in a patient with confirmed inherited hemophilia B.

Muscle bleeding in hemophilia can present in various ways such as swelling, stiffness, or pain. It is important to keep this in mind, as prompt recognition and initiation of treatment with factor infusions are key. A factor-level activity of at least 50% is needed in severe muscle hematomas. Severe bleeds may require multiple factor infusions as well. Factor IX transfusions can be dosed based on prior experience with a known patient. In the case of new patients, approximately 50-60 international units/kg of factor IX can be given to raise the factor IX level by 50%. Some cases have reported treatment with the addition of immunosuppressive agents such as cyclophosphamide and prednisone [9,10], as well as antibody therapy with emicizumab [8] and rituximab [11]. Patients should be monitored for any drops in hemoglobin as well as signs of neurovascular bundle impingement. If there is a worsening of the bleed or any complications that arise despite medical therapy, the patient can then be evaluated for surgical decompression [7].

## Conclusions

Hematomas can occur in atypical locations, such as the lateral chest wall, and a high degree of suspicion should be maintained especially in hemophiliacs. A lateral chest wall hematoma should be considered in the differential diagnosis for patients presenting with back pain. The absence of trauma to the area should not deter additional investigation, as prompt intervention is essential to avoid complications.

## References

[1] Konkle BA, Nakaya FS (1993-2023). Hemophilia B. https://www.ncbi.nlm.nih.gov/books/NBK1404/?report=reader.

[2] Bevan P, Menon A, Bunton R (2014). Spontaneous chest wall hematoma with dual antiplatelet therapy. Can J Cardiol.

[3] Dwebi Dwebi, M. M., Cumber Cumber, P P (2019). Spontaneous chest wall haematoma in chronic myeloid leukaemia. Egypt J Intern Med.

[4] Miyamoto K, Suzuki K, Nakamura M (2021). Hypovolemic shock induced by a large chest wall hematoma caused by a single rib fracture in an elderly patient. Trauma Case Rep.

[5] Choudhry MS, Sultan A, Hassan M, Ali M, Zaidi SM (2020). Right scapular swelling revealed to be a spontaneous lateral chest wall hematoma: an intriguing case report. Cureus.

[6] Aviña-Zubieta A, Galindo-Rodriguez G, Lavalle C (1998). Rheumatic manifestations of hematologic disorders. Curr Opin Rheumatol.

[7] Hoots Hoots, K K, Shapiro A (2023). Clinical manifestations and diagnosis of hemophiliae. UpToDate.

[8] Al-Banaa K, Alhillan A, Hawa F (2019). Emicizumab use in treatment of acquired hemophilia A: a case report. Am J Case Rep.

[9] Miller K, Neely JE, Krivit W, Edson JR (1978). Spontaneously acquired factor IX inhibitor in a nonhemophiliac child. J Pediatr.

[10] Raval M, Kallamadi R, Bande D (2012). A rare case of acquired hemophilia A associated with myelodysplastic syndrome. Int J Clin Exp Med.

[11] Fortier JC, Pang SS, Amofa-Ho S, Harris NS, Zumberg M (2022). A case of acquired hemophilia A and congenital hemophilia B. Cureus.

